# Phenytoin toxicity due to fluoropyrimidines (5FU/capecitabine): three case reports

**DOI:** 10.1038/sj.bjc.6601137

**Published:** 2003-08-12

**Authors:** K Brickell, D Porter, P Thompson

**Affiliations:** 1 Oncology Department, Auckland Hospital, Private Bag 92024, Auckland 1, New Zealand

**Keywords:** phenytoin, fluoropyrimidine, fluorouracil, capecitabine

## Abstract

Interactions between phenytoin and the fluoropyrimidine derivatives, 5 fluorouracil (5FU) and capecitabine, are not commonly documented or widely recognised. As more people with significant medical comorbidities are now being considered for chemotherapy, it is increasingly likely that people receiving phenytoin will be prescribed treatment with fluoropyrimidines. We present two cases of clinical phenytoin toxicity occurring after starting treatment with 5-fluorouracil/folinic acid (5FU/FA) and a third who developed clinical phenytoin toxicity after starting capecitabine (an orally administered prodrug of fluorouracil).

## CASE I

A 65-year-old Asian man, with a history of more than 10 years of idiopathic generalised tonic clonic epilepsy, had been treated with a stable dose of phenytoin (300 mg daily) and phenobarbitone (90 mg daily). He was diagnosed with Dukes' B adenocarcinoma of the colon in 1999. He had recurrence of the disease in 2002 with an intra-abdominal mass, para-aortic lymphadenopathy and pulmonary metastases. He was started on palliative 5FU/FA at doses of 370 and 20 mg m^−2^ weekly. His past medical history was unremarkable and his other medications were morphine, lactulose, coloxyl and senna, multivitamins, and loratadine. His liver enzymes were normal.

Seven weeks after starting chemotherapy he was admitted with a 10-day history of progressive confusion, drowsiness, generalised weakness, and fatigue preventing him from walking. On examination he was drowsy, dysarthric, had bilateral gaze evoked nystagmus and marked limb ataxia. He was unable to stand unsupported. The serum phenytoin level was 162 *μ*mol l^−1^ (therapeutic level 40–80 *μ*mol l^−1^). His phenobarbitone level was 89 *μ*mol l^−1^ (therapeutic level 65–170 *μ*mol l^−1^).

The phenytoin was withheld for 5 days until his serum level was less than 80 *μ*mol l^−1^. His symptoms and signs resolved with the falling serum phenytoin level. He was restarted on a reduced dose of phenytoin (230 mg daily) when his serum level was 67 *μ*mol l^−1^. On this dose, his serum phenytoin level initially increased to 75 *μ*mol l^−1^ before dropping to 22 *μ*mol l^−1^ 5 weeks after stopping chemotherapy. The chemotherapy was stopped after two cycles due to progression of the disease.

## CASE 2

The second case is of a 60-year-old man with a history of post-traumatic generalised tonic clonic epilepsy caused by a head injury in 1988. He was treated with phenytoin 430 mg daily (200 mg mane, 230 mg nocte) with stable phenytoin serum levels (75 *μ*mol l^−1^ on 17.11.2000) and no symptoms of phenytoin toxicity since that time. He was on no other medications.

He was diagnosed with Dukes' C adenocarcinoma of the transverse colon and was started on adjuvant 5 FU/FA with the same protocol as patient one.

At 4 weeks after starting chemotherapy, he presented with a 6-day history of progressive light-headedness and inability to stand unsupported. On examination he had severe gait ataxia and was unable to walk. His mental state and other neurological signs were not documented. His phenytoin level was 213 *μ*mol l^−1^. His phenytoin was withheld for 7 days, until his level had come down to 42 *μ*mol l^−1^ when he was restarted on a reduced dose (300 mg daily). At this dose of phenytoin his serum level rose from 42 to 118 *μ*mol l^−1^. His dose of phenytoin was further reduced and he was discharged on 100 mg phenytoin daily with ongoing chemotherapy. Since discharge, he has had a seizure associated with a subtherapeutic serum phenytoin level of 24 *μ*mol l^−1^ and has had his phenytoin dose increased to 200 mg day^−1^.

## CASE 3

The last case is of a 45-year-old woman with a greater than 10-year history of poorly controlled generalised tonic clonic epilepsy requiring three anticonvulsants: phenytoin 400 mg daily (200 mg twice a day); clobazam 10 mg nocte; and sodium valproate 1 gm twice daily. Serum phenytoin levels fluctuated at this dose and the last phenytoin level, 18 months before admission, was 141 *μ*mol l^−1^. There are no levels available between then and when she was admitted. Her other medications were ranitidine, morphine, paracetamol, and fluticasone propionate.

She developed breast cancer in 2001 and underwent adjuvant treatment with intravenous cyclophosphamide, methotrexate, and 5 FU (CMF) chemotherapy without clinical evidence of phenytoin toxicity. Her cancer progressed and she commenced on palliative chemotherapy (single-agent doxorubicin, followed by docetaxel, and finally twice daily capecitabine, completing two cycles). The protocol used for CMF was: cyclophosphamide 100 mg m^−2^ day 1–14, methotrexate 40 mg m^−2^ days 1 and 8, 5 FU 600 mg m^−2^ days 1 and 8 in a 4 weekly cycle and capecitabine was prescribed at a dose of 1500 mg twice daily for 14 days, repeating 3 weekly.

The first cycle of capecitabine was one-third (500 mg twice a day) her prescribed dose due to patient misunderstanding, but her second dose was at the intended dose (1500 mg twice a day).

Nearly 6 weeks after starting this therapy (just over 2 weeks after the full therapeutic dose), she was admitted 2-day history of an unsteady gait, recurrent falls, weakness, poor balance, and light-headedness. On examination she had dysarthria, poor balance, and limb ataxia. Her serum phenytoin level was 161 *μ*mol l^−1^ (therapeutic level 40–80 *μ*mol l^−1^). Her phenytoin was withheld for 5 days until her serum phenytoin level was under 80 *μ*mol l^−1^ and the dose was reduced to 300 mg daily. Her serum phenytoin level increased on this reduced dose (level=104 *μ*mol l^−1^) and she became light-headed again. She was finally discharged on 260 mg of phenytoin daily. After 2 months, her serum phenytoin level fell to 18 *μ*mol l^−1^ while remaining at this dose. Her capecitabine was stopped after two cycles due to progressive disease.

## DISCUSSION

Our three cases add to the small volume of literature suggesting that there is a genuine interaction between 5FU and phenytoin. This hypothesis is supported by the difficulty in maintaining a nontoxic serum phenytoin level in our patients, despite reductions in the daily dose of phenytoin during treatment. Further support for the interaction between these medications was the fall in phenytoin levels once the chemotherapy was discontinued, which required ongoing phenytoin dose adjustments. This observation provides further evidence of a drug interaction, which resolves after treatment ends.

There are two case reports in the literature about interactions between phenytoin and 5FU resulting in phenytoin toxicity and one case report of two breast cancer patients who developed phenytoin toxicity after capecitabine (Schaller, 2000; Gilbar and Brodribb, 2001; Rosemergy and Findlay, 2002).

In accordance with these earlier reports, it seems unlikely that the toxicity in our first two patients can be attributed to alterations in phenytoin binding, absorption, sampling times for drug levels, or changes in compliance. The first two patients had been on a stable dose of phenytoin for more than 10 years without evidence of phenytoin toxicity. Neither patient had significant abnormalities in their liver enzymes or serum albumin to explain altered phenytoin binding or clearance. In the third patient, the relationship between her chemotherapy and serum phenytoin level is not as definite as there are many other potential factors that could have attributed to her elevated phenytoin level; in particular, her past history of elevated phenytoin levels, long-standing cognitive impairment, and hypoalbuminaemia. The observation that her serum phenytoin level remained unstable despite a reduced dose in hospital and supervised dispensing, suggests that capecitabine had indeed altered the metabolism of phenytoin rather than it being secondary to difficulties with compliance.

Phenytoin is metabolised in the liver, mainly by the CYP2C9 isoenzyme. A small amount is metabolised by the CYP2C19 isotype (Ninomiya *et al*, 2000; Brandolese *et al*, 2001). Fluorouracil may competitively inhibit the clearance of phenytoin by the CYP2C9 isoenzyme or alternatively, it may reduce its synthesis (Levy, 1995, Gilbar and Brodribb, 2001), thereby explaining a delay in the occurrence of toxicity.

Increased phenytoin plasma concentrations have been reported during concomitant use of capecitabine, but formal interaction studies have not been carried out. The mechanism of interaction is thought to be at the level of the CYP2C9 isoenzyme system (Roche Xeloda (capecitabine) data sheet), in accordance with its role as a prodrug for 5 FU (van Cutsem and Peeters, 1999; Ratain 2002; Wagstaff *et al*, 2003).

## CONCLUSION

The risk of phenytoin toxicity with concurrent 5 FU administration appears real. The combined evidence of these and previous cases suggest that further investigations into how these medications interact at the cytochrome isoenzyme level are warranted. In the meantime, it is prudent to monitor for clinical manifestations of phenytoin toxicity and perform more frequent phenytoin levels in all patients receiving phenytoin and either 5 FU or capecitabine. Monitoring should continue after the chemotherapy has finished to ensure that the phenytoin level is sufficient to prevent seizures.
